# Supplementary material for: Modeling glioblastoma heterogeneity as a dynamic network of cell states
Source: Mol Syst Biol. 2021 Sep 16;17(9):e10105. doi: 10.15252/msb.202010105 (PMC8444284; doi:10.15252/msb.202010105)
Supplement: Supplementary file 6 — Source Data for Figure 5 [file MSB-17-e10105-s004.zip › Figure5A_sourcedata/GSEA_3017/nosigdb_stateE.GseaPreranked.1622795031093/RICHARDS_NOWAKOWSKI_SCIENCE_2017_RG_EARLY_UPREG.html]

Details for gene set RICHARDS\_NOWAKOWSKI\_SCIENCE\_2017\_RG\_EARLY\_UPREG[GSEA]

|  || Dataset | state63017 |
| Phenotype | NoPhenotypeAvailable |
| Upregulated in class | na\_neg |
| GeneSet | RICHARDS\_NOWAKOWSKI\_SCIENCE\_2017\_RG\_EARLY\_UPREG |
| Enrichment Score (ES) | -0.6663411 |
| Normalized Enrichment Score (NES) | -3.6744568 |
| Nominal p-value | 0.0 |
| FDR q-value | 0.0 |
| FWER p-Value | 0.0 |
Table: GSEA Results Summary

  

Fig 1: Enrichment plot: RICHARDS\_NOWAKOWSKI\_SCIENCE\_2017\_RG\_EARLY\_UPREG      
 Profile of the Running ES Score & Positions of GeneSet Members on the Rank Ordered List

  

| PROBE | GENE SYMBOL | GENE\_TITLE | RANK IN GENE LIST | RANK METRIC SCORE | RUNNING ES | CORE ENRICHMENT || 1 | TTYH1 |  |  | 19 | 0.731 | -0.0153 | No |
| 2 | MAP1B |  |  | 22 | 0.715 | -0.0099 | No |
| 3 | CNN3 |  |  | 24 | 0.702 | -0.0035 | No |
| 4 | C1orf61 |  |  | 43 | 0.553 | -0.0195 | No |
| 5 | ATF3 |  |  | 101 | 0.397 | -0.0853 | No |
| 6 | TPM1 |  |  | 108 | 0.388 | -0.0884 | No |
| 7 | ZFP36L2 |  |  | 162 | 0.324 | -0.1500 | No |
| 8 | FTH1 |  |  | 204 | 0.293 | -0.1973 | No |
| 9 | ITGB8 |  |  | 239 | 0.268 | -0.2361 | No |
| 10 | GPC4 |  |  | 246 | 0.264 | -0.2406 | No |
| 11 | FDFT1 |  |  | 266 | 0.252 | -0.2612 | No |
| 12 | ZFP36L1 |  |  | 287 | -0.255 | -0.2830 | No |
| 13 | RAN |  |  | 298 | -0.257 | -0.2925 | No |
| 14 | AHI1 |  |  | 310 | -0.261 | -0.3031 | No |
| 15 | HMGA1 |  |  | 313 | -0.261 | -0.3027 | No |
| 16 | TGIF1 |  |  | 318 | -0.263 | -0.3048 | No |
| 17 | PSIP1 |  |  | 319 | -0.263 | -0.3019 | No |
| 18 | CNTLN |  |  | 326 | -0.264 | -0.3064 | No |
| 19 | HSPD1 |  |  | 329 | -0.264 | -0.3059 | No |
| 20 | RPL22L1 |  |  | 350 | -0.269 | -0.3276 | No |
| 21 | SNRPG |  |  | 398 | -0.283 | -0.3823 | No |
| 22 | LSM4 |  |  | 406 | -0.284 | -0.3878 | No |
| 23 | SIVA1 |  |  | 412 | -0.286 | -0.3908 | No |
| 24 | PPA1 |  |  | 420 | -0.289 | -0.3962 | No |
| 25 | MCM4 |  |  | 457 | -0.301 | -0.4372 | No |
| 26 | SNRPE |  |  | 458 | -0.302 | -0.4339 | No |
| 27 | CBFB |  |  | 500 | -0.314 | -0.4808 | No |
| 28 | PIK3R3 |  |  | 508 | -0.317 | -0.4860 | No |
| 29 | BARD1 |  |  | 510 | -0.318 | -0.4837 | No |
| 30 | RHEB |  |  | 528 | -0.323 | -0.5011 | No |
| 31 | CEP135 |  |  | 532 | -0.325 | -0.5012 | No |
| 32 | SNHG1 |  |  | 538 | -0.328 | -0.5038 | No |
| 33 | DCLRE1C |  |  | 544 | -0.330 | -0.5063 | No |
| 34 | CDC25C |  |  | 549 | -0.332 | -0.5076 | No |
| 35 | MCM3 |  |  | 560 | -0.340 | -0.5161 | No |
| 36 | HMGB3 |  |  | 577 | -0.348 | -0.5320 | No |
| 37 | CACYBP |  |  | 589 | -0.352 | -0.5416 | No |
| 38 | DDX39A |  |  | 590 | -0.353 | -0.5378 | No |
| 39 | BTG3 |  |  | 615 | -0.365 | -0.5633 | No |
| 40 | RPS24 |  |  | 635 | -0.381 | -0.5825 | No |
| 41 | ZEB1 |  |  | 653 | -0.396 | -0.5990 | No |
| 42 | PBX3 |  |  | 662 | -0.403 | -0.6045 | No |
| 43 | PGAM1 |  |  | 670 | -0.410 | -0.6086 | No |
| 44 | KIF18A |  |  | 673 | -0.412 | -0.6065 | No |
| 45 | NUP50 |  |  | 677 | -0.415 | -0.6056 | No |
| 46 | CHEK1 |  |  | 704 | -0.433 | -0.6329 | No |
| 47 | TIMELESS |  |  | 707 | -0.435 | -0.6305 | No |
| 48 | RBBP8 |  |  | 714 | -0.440 | -0.6331 | No |
| 49 | TTK |  |  | 724 | -0.454 | -0.6392 | No |
| 50 | SPAG5 |  |  | 729 | -0.458 | -0.6391 | No |
| 51 | KNSTRN |  |  | 751 | -0.485 | -0.6596 | No |
| 52 | PRR11 |  |  | 755 | -0.488 | -0.6579 | No |
| 53 | CDCA3 |  |  | 759 | -0.497 | -0.6562 | No |
| 54 | GMNN |  |  | 766 | -0.510 | -0.6579 | No |
| 55 | RTKN2 |  |  | 767 | -0.511 | -0.6523 | No |
| 56 | KIF5B |  |  | 770 | -0.515 | -0.6491 | No |
| 57 | CEP152 |  |  | 785 | -0.542 | -0.6604 | Yes |
| 58 | FANCD2 |  |  | 787 | -0.544 | -0.6556 | Yes |
| 59 | HAUS8 |  |  | 792 | -0.560 | -0.6544 | Yes |
| 60 | HMGN2 |  |  | 795 | -0.564 | -0.6507 | Yes |
| 61 | RACGAP1 |  |  | 796 | -0.566 | -0.6445 | Yes |
| 62 | HMGB1 |  |  | 797 | -0.567 | -0.6382 | Yes |
| 63 | CENPN |  |  | 805 | -0.574 | -0.6405 | Yes |
| 64 | USP1 |  |  | 806 | -0.575 | -0.6342 | Yes |
| 65 | DEK |  |  | 812 | -0.591 | -0.6339 | Yes |
| 66 | MND1 |  |  | 821 | -0.615 | -0.6370 | Yes |
| 67 | CKAP2L |  |  | 826 | -0.625 | -0.6350 | Yes |
| 68 | HELLS |  |  | 830 | -0.635 | -0.6318 | Yes |
| 69 | PCNA |  |  | 832 | -0.640 | -0.6260 | Yes |
| 70 | CENPW |  |  | 835 | -0.643 | -0.6214 | Yes |
| 71 | CDKN3 |  |  | 836 | -0.653 | -0.6142 | Yes |
| 72 | ITGB3BP |  |  | 838 | -0.661 | -0.6082 | Yes |
| 73 | ANP32E |  |  | 841 | -0.676 | -0.6032 | Yes |
| 74 | KIF22 |  |  | 842 | -0.678 | -0.5957 | Yes |
| 75 | BIRC5 |  |  | 843 | -0.679 | -0.5883 | Yes |
| 76 | BRCA1 |  |  | 846 | -0.691 | -0.5832 | Yes |
| 77 | ZWINT |  |  | 847 | -0.692 | -0.5756 | Yes |
| 78 | SPC24 |  |  | 848 | -0.694 | -0.5679 | Yes |
| 79 | ECT2 |  |  | 854 | -0.717 | -0.5662 | Yes |
| 80 | KIF2C |  |  | 855 | -0.721 | -0.5583 | Yes |
| 81 | CENPK |  |  | 857 | -0.722 | -0.5516 | Yes |
| 82 | SYNE2 |  |  | 859 | -0.728 | -0.5448 | Yes |
| 83 | ANLN |  |  | 860 | -0.733 | -0.5368 | Yes |
| 84 | KIF4A |  |  | 861 | -0.736 | -0.5287 | Yes |
| 85 | CCNB2 |  |  | 865 | -0.745 | -0.5242 | Yes |
| 86 | ARHGAP11A |  |  | 867 | -0.756 | -0.5171 | Yes |
| 87 | NDC80 |  |  | 869 | -0.762 | -0.5100 | Yes |
| 88 | BUB1B |  |  | 871 | -0.766 | -0.5028 | Yes |
| 89 | CCNA2 |  |  | 872 | -0.770 | -0.4944 | Yes |
| 90 | KIF23 |  |  | 873 | -0.772 | -0.4859 | Yes |
| 91 | KIF20B |  |  | 876 | -0.779 | -0.4798 | Yes |
| 92 | CKAP2 |  |  | 878 | -0.781 | -0.4725 | Yes |
| 93 | NCAPG2 |  |  | 879 | -0.781 | -0.4639 | Yes |
| 94 | PTTG1 |  |  | 880 | -0.791 | -0.4552 | Yes |
| 95 | KIF14 |  |  | 885 | -0.809 | -0.4512 | Yes |
| 96 | EZH2 |  |  | 886 | -0.810 | -0.4423 | Yes |
| 97 | KIF15 |  |  | 890 | -0.834 | -0.4369 | Yes |
| 98 | H2AFZ |  |  | 893 | -0.849 | -0.4300 | Yes |
| 99 | RAD51AP1 |  |  | 894 | -0.853 | -0.4206 | Yes |
| 100 | DBF4 |  |  | 896 | -0.864 | -0.4124 | Yes |
| 101 | SMC2 |  |  | 897 | -0.867 | -0.4028 | Yes |
| 102 | CENPE |  |  | 899 | -0.873 | -0.3945 | Yes |
| 103 | PBK |  |  | 900 | -0.880 | -0.3848 | Yes |
| 104 | SMC4 |  |  | 902 | -0.886 | -0.3763 | Yes |
| 105 | DUT |  |  | 903 | -0.887 | -0.3666 | Yes |
| 106 | MIS18BP1 |  |  | 904 | -0.889 | -0.3568 | Yes |
| 107 | CCNB1 |  |  | 906 | -0.895 | -0.3482 | Yes |
| 108 | CKS1B |  |  | 909 | -0.912 | -0.3407 | Yes |
| 109 | CKS2 |  |  | 911 | -0.922 | -0.3318 | Yes |
| 110 | KIF11 |  |  | 913 | -0.957 | -0.3225 | Yes |
| 111 | TYMS |  |  | 917 | -0.965 | -0.3156 | Yes |
| 112 | CENPU |  |  | 918 | -0.971 | -0.3049 | Yes |
| 113 | PRC1 |  |  | 919 | -0.980 | -0.2942 | Yes |
| 114 | MAD2L1 |  |  | 921 | -0.990 | -0.2845 | Yes |
| 115 | UBE2T |  |  | 922 | -0.994 | -0.2736 | Yes |
| 116 | DLGAP5 |  |  | 923 | -0.998 | -0.2626 | Yes |
| 117 | SPC25 |  |  | 924 | -1.033 | -0.2513 | Yes |
| 118 | HIST1H1E |  |  | 925 | -1.049 | -0.2398 | Yes |
| 119 | UBE2C |  |  | 928 | -1.108 | -0.2301 | Yes |
| 120 | NUSAP1 |  |  | 929 | -1.112 | -0.2179 | Yes |
| 121 | TPX2 |  |  | 930 | -1.124 | -0.2055 | Yes |
| 122 | NCAPG |  |  | 931 | -1.142 | -0.1930 | Yes |
| 123 | ASPM |  |  | 932 | -1.146 | -0.1804 | Yes |
| 124 | ATAD2 |  |  | 933 | -1.162 | -0.1676 | Yes |
| 125 | CENPF |  |  | 935 | -1.200 | -0.1557 | Yes |
| 126 | HIST1H1C |  |  | 936 | -1.220 | -0.1423 | Yes |
| 127 | HMGB2 |  |  | 937 | -1.238 | -0.1287 | Yes |
| 128 | CDK1 |  |  | 938 | -1.248 | -0.1150 | Yes |
| 129 | RRM2 |  |  | 940 | -1.403 | -0.1008 | Yes |
| 130 | MKI67 |  |  | 941 | -1.407 | -0.0853 | Yes |
| 131 | TOP2A |  |  | 942 | -1.417 | -0.0698 | Yes |
| 132 | HIST1H1D |  |  | 944 | -1.810 | -0.0511 | Yes |
| 133 | HIST1H1B |  |  | 945 | -1.903 | -0.0302 | Yes |
| 134 | HIST1H4C |  |  | 946 | -2.752 | -0.0000 | Yes |
Table: GSEA details [plain text format]

  

Fig 2: RICHARDS\_NOWAKOWSKI\_SCIENCE\_2017\_RG\_EARLY\_UPREG: Random ES distribution      
 Gene set null distribution of ES for **RICHARDS\_NOWAKOWSKI\_SCIENCE\_2017\_RG\_EARLY\_UPREG**
